# Fine-tuning of NADH oxidase decreases byproduct accumulation in respiration deficient xylose metabolic *Saccharomyces cerevisiae*

**DOI:** 10.1186/1472-6750-14-13

**Published:** 2014-02-14

**Authors:** Jin Hou, Fan Suo, Chengqiang Wang, Xiaowei Li, Yu Shen, Xiaoming Bao

**Affiliations:** 1 State Key Laboratory of Microbial Technology, Shandong University, Shanda Nan Road 27, Jinan 250100, China

**Keywords:** NADH oxidase, Xylose metabolism pathways, Cofactor, Glycerol, Xylitol

## Abstract

**Background:**

Efficiently utilizing all available carbon from lignocellulosic feedstock presents a major barrier to the production of economically feasible biofuel. Previously, to enable xylose utilization, we introduced a cofactor-dependent xylose reductase (XR) and xylitol dehydrogenase (XDH) pathway, or a cofactor-independent xylose isomerase (XI) pathway, into *Saccharomyces cerevisiae*. The resulting strains metabolized xylose with high efficiency. However, in both pathway recombinant strains, the cofactor imbalance caused accumulation of the byproducts glycerol and/or xylitol and reduced the ethanol production efficiency.

**Results:**

In this study, we introduced NADH oxidase from *Lactococcus lactis* into both XI and XR-XDH pathway recombinant strains. To reduce byproduct accumulation while maintaining xylose metabolism, we optimized the expression level of NADH oxidase by comparing its expression under the control of different promoters and plasmids. In recombinant XI strains, NADH oxidase was expressed at different levels, regulated by the *GPD2* promoter or *TEF1* promoter in the 2 μ plasmid. The expression under the control of *GPD2* promoter decreased glycerol production by 84% and increased the ethanol yield and specific growth rate by 8% and 12%, respectively. In contrast, in the recombinant XR-XDH strains, such expression level was not efficient enough to decrease the byproduct accumulation. Therefore, higher NADH oxidase expression levels were tested. In the strain expressing NADH oxidase under the control of the *TEF1* promoter in the centromeric plasmids, xylitol and glycerol production were reduced by 60% and 83%, respectively, without significantly affecting xylose consumption.

**Conclusions:**

By fine-tuning NADH oxidase expression, we decreased the glycerol or/and xylitol production in both recombinant XI and XR-XDH xylose-metabolizing yeast strains. The optimal NADH oxidase expression levels depend on metabolic pathways. Similar cofactor engineering strategies could maximize the production of other redox dependent metabolites.

## Background

**Figure 1:**
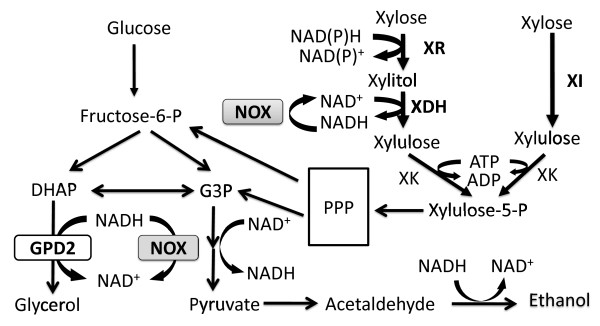
**Glucose and xylose metabolic pathways in recombinant *S. cerevisiae*.** The introduction NADH oxidase can reduce the amount of excess NADH which is normally produced by glycerol 3-phosphate dehydrogenase (GPD) or xylitol dehydrogenase (XDH).

Efficient utilization of all available carbon from lignocellulosic feedstock presents a major barrier to economical biofuel production [1]. Xylose is the second predominant sugar in lignocellulosic feedstock after glucose. However, *Saccharomyces cerevisiae*, which is ubiquitously employed in ethanol production, cannot naturally metabolize xylose. Consequently, throughout the past few decades, the introduction of xylose metabolic pathways into *S. cerevisiae* has been extensively researched [2,3]. Two pathways have been studied widely for D-xylose utilization. In fungi and xylose-metabolic yeasts, D-xylose is reduced to xylitol by NAD(P)H-dependent xylose reductase (XR), encoded by *XYL1* and xylitol is then oxidized to D-xylulose by NAD^+^-dependent xylitol dehydrogenase (XDH), encoded by *XYL2*[4,5]. The resulting D-xylulose is converted to xylulose-5-phosphate by endogenous xylulose kinase (XK). Alternatively, some bacteria and fungi can directly convert D-xylose to xylulose via the cofactor-independent xylose isomerase (XI) pathway. Both pathways have been successfully introduced into *S. cerevisiae*, allowing the recombinant strains to produce ethanol from xylose (Figure 1) [6-11]. Xylose metabolism has been improved by over-expressing the endogenous xylulose kinase genes and the genes involved in the non-oxidative pentose phosphate pathway [12,13].

Introducing the *Scheffersomyces stipitis* XR-XDH pathway into *S. cerevisiae* has enabled the yeast to effectively utilize xylose [4,5,14]. Our previous work also demonstrated efficient xylose uptake by strains expressing this pathway [12,15]. However, the different cofactor dependence of XR and XDH leads to cofactor imbalance and xylitol accumulation. Recent studies have focused on metabolic engineering to balance intracellular cofactor levels or on modifying the cofactor specificities of XR or XDH to establish an oxidation-reduction cycle [16-20]. Several strategies have been implemented for balancing intracellular cofactors in recombinant *S. cerevisiae*. These include manipulating ammonia assimilation from being NADPH dependent to being NADH dependent by replacing *GDH1* (which encodes NADPH-dependent glutamate dehydrogenase) with *GDH2* (which encodes NADH-dependent glutamate dehydrogenase), expressing the *Kluyveromyces lactis GDP1* gene, which encodes a fungal NADP^+^-dependent D-glyceraldehyde-3-phosphate dehydrogenase, expressing the *gapN* gene from *Streptococcus* mutants, which encodes a non-phosphorylating NADP^+^-dependent *GAPDH*, and over-expressing the truncated *POS5* gene, which encodes cytosolic NADH kinase [15,19,21,22].

An alternative pathway for xylose catabolism is the isomerase-based pathway. This pathway is cofactor independent, and therefore could lead to higher theoretical ethanol yields. However, only a few xylose isomerase genes have been successfully expressed in *S. cerevisiae*, derived from organisms such as *Piromyces sp.*[6,23], *Orpinomyces sp.*[24], *Clostridium phytofermentans*[25] and *Prevotella ruminicola*[26]. Further engineering strategies, such as adaptive evolutionary engineering [8] and over-expressing downstream pathways [7,27], have been implemented to improve xylose consumption and cell growth.

Recently, we obtained a new XI (*xylA*) gene from bovine rumen. The activity of this XI in *S. cerevisiae* was slightly higher than the XI from *Piromyces sp.*[28]. We also engineered the host strain to over-express the endogenous xylulose kinase gene (*XKS1*) and the genes in non-oxidative pentose phosphate pathway, eliminating the respiration by deleting cytochrome C oxidase subunit IV encoding gene *COX4*, and adaptive evolution [13]. The recombinant strain showed high xylose metabolism capability and high ethanol yield under both aerobic and anaerobic conditions. However, although no xylitol was accumulated, substantial amounts of glycerol were produced as the major byproduct [28].

In industrial ethanol processes, up to 4% of the sugar feedstock is converted into glycerol by *S. cerevisiae*, which is an unwanted loss of carbon source [29,30]. Glycerol synthesis plays important roles in yeast osmoregulation and in regulating intracellular redox balance [31]. It is produced from dihydroxyacetone phosphate (DHAP) through the catalysis of glycerol-3-phosphate dehydrogenase (GPD, encoded by genes *GPD1* and *GPD2*) and glycerol-3-phosphate phosphatase (GPP, encoded by genes *GPP1* and *GPP2*). Researchers have expended considerable effort in minimizing glycerol formation. One approach is to delete one or both of *GPD1* and *GPD2* as well as the genes involved in glycerol transport, such as *FPS1*[32,33]. Because cells lacking the *GPD1* and *GPD2* genes cannot grow anaerobically, the promoter of *GPD1* has been engineered in *GPD2* deletion background [34]. Alternative approaches aim at manipulating the redox cofactor metabolism to reduce cytosolic NADH accumulation [35]. For example, Nissen *et al.* deleted *GDH1* (encoding NADPH-dependent glutamate dehydrogenase), while overexpressing *GLN1* and *GLT1* (encoding glutamine synthetase and glutamate synthase, respectively). Their engineered strains demonstrated reduced glycerol yield and increased ethanol yield [36]. Guo *et al*. simultaneously deleted *GPD1* and introduced the non-phosphorylating NADP^+^-dependent *GAPDH* gene *gapN* into strains overexpressing the trehalose synthesis genes *TPS1* and *TPS2*, thereby obtaining a high ethanol-yielding strain [32]. Zhang *et al*. combined the expression of NADP^+^-dependent *GAPDH* gene *gapN* with either a NAD^+^-dependent fumarate reductase gene *frdA*, or an acetylating NAD^+^-dependent acetaldehyde dehydrogenase for reoxidizing NADH [37].

Water-forming NADH oxidase can oxidize cytosolic NADH to NAD^+^, accompanied by a reduction of O_2_ to H_2_O, when oxygen is available. Previous studies have demonstrated the capability of NADH oxidase expression to reduce xylitol production during xylose metabolism [20], but in this approach, aerobic cultivation combined with oxygen-limited fermentation has to be performed to supply oxygen for NADH oxidase, which results in low ethanol production. However, if the ethanol yield is enhanced by purely anaerobic cultivation, the NADH oxidase reaction is deprived of its required oxygen. In our previous work, we demonstrated that respiration-deficient xylose-metabolizing strains can efficiently produce ethanol from xylose and glucose under both aerobic and anaerobic conditions [12]. Therefore, in the present study, we expressed water-forming NADH oxidase derived from *Lactococcus lactis* in our respiration-deficient xylose-metabolizing strains (Figure 1). The fermentation process is aerobically controlled to supply oxygen for NADH oxidase without compromising ethanol production. The impact on byproduct accumulation and ethanol production was studied in both recombinant XI strains and recombinant XR-XDH strains. To decrease the byproduct accumulation without affecting yeast growth and sugar metabolism, different NADH oxidase expression levels were compared by expressing the *noxE* gene controlled by different promoters in the 2 μ or centromeric plasmids under glucose and xylose co-cultivation conditions.

## Results

### NADH oxidase expression decreases glycerol production in recombinant XI strains

**Figure 2:**
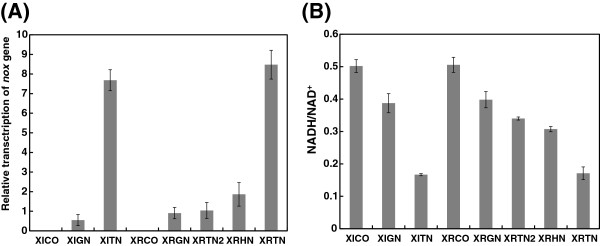
**The evaluation of the transcription and expression of *noxE *gene in the recombinant strains.** Relative transcription of *noxE* gene **(A**) and intracellular concentrations of NADH/NAD^+^ ratio **(B)** in the recombinant strains. Measurements are the average value ± standard error from independent duplicate experiments.

As mentioned previously, glycerol is the main byproduct of xylose metabolism in recombinant XI strains [7]. We therefore aimed to suppress glycerol production by expressing the NADH oxidase gene. Two different NADH oxidase expression levels were selected under the control of either the *TEF1* or *GPD2* promoter in the 2 μ plasmid. The relative transcription of *noxE* in XITN was about 13 fold higher than that in XIGN (Figure 2A). The specific enzyme activities of NADH oxidase in XITN and XIGN were 1.85 and 0.08 U mg^-1^ protein, respectively (Table 1). As a consequence of NADH oxidase expression, the intracellular NADH/NAD^+^ ratio decreased by 67% and 23% in XITN and XIGN, respectively, relative to XICO (Figure 2B).

**Table 1:** **NADH oxidase activities in recombinant strains of *S. cerevisiae***

**Strain**	**Description**	**NADH oxidase activity (U mg**^**-1 **^**total protein)**
XICO	XI, pYX242-WS	0
XIGN	XI, pYX242-GPD2nox	0.08 ± 0.01
XITN	XI, pYX242-TEF1nox	1.85 ± 0.06
XRCO	XR-XDH, pYX242-WS	0
XRGN	XR-XDH, pYX242-GPD2nox	0.05 ± 0.00
XRTN2	XR-XDH, pRS315-TEF1nox	0.17 ± 0.00
XRHN	XR-XDH, pYX242-HXK2nox	0.57 ± 0.03
XRTN	XR-XDH, pYX242-TEF1nox	1.92 ± 0.12

**Figure 3:**
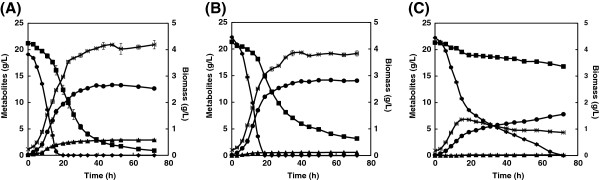
**Aerobic batch cultivations of recombinant XI strains with a mixture of 20 g/L glucose and 20 g/L xylose.** The experiments were performed in duplicate. **(A)**, XICO; **(B)**, XIGN; and **(C)**, XITN. Symbols: ◆, glucose; ■, xylose; ●, ethanol; ▲, glycerol; ×, biomass. Measurements are the average value ± standard error from independent duplicate cultivations.

We then studied the impact of NADH oxidase expression on glucose and xylose co-cultivations. As shown in Figure 3 and Table 2, expressing NADH oxidase under the strong constitutive *TEF1* promoter in the 2 μ plasmid completely suppressed glycerol production, but also significantly reduced glucose and xylose metabolism, and decreased the ethanol yield by about 17% (relative to the control strain). This may be because the strong NADH oxidase expression in XITN depleted the pool of NADH (Figure 2B). When NADH oxidase was regulated by the *GPD2* promoter in the 2 μ plasmid, the glycerol yield reduced by 84% in XIGN, while the ethanol yield and specific growth rate increased by 8% and 12%, respectively (Figure 3B and Table 2). The biomass yield also increased slightly, without significantly affecting the xylose consumption. These results show that expressing NADH oxidase under the control of the *GPD2* promoter in recombinant XI strains can effectively reduce glycerol production and increase the ethanol yield.

**Table 2:** **Physiological parameters of recombinant *S. cerevisiae *** **strains from batch cultivations on glucose and xylose**

**Strain**	**μ**_**max **_**(h**^**-1**^**)**^**a**^	**Consumed xylose in 72 h (g L**^**-1**^**)**	**Sugar consumption rate (g L**^**-1**^ **h**^**-1**^**)**^**b**^		**Yield on sugars (g g**^**-1**^**)**^**c**^			**Carbon balance**
			**Glucose**	**Xylose**	**Biomass**	**Ethanol**	**Glycerol**	
XICO	0.17	20.14	1.34	0.48	0.10	0.35	0.084	0.92
XIGN	0.19	18.81	1.33	0.46	0.12	0.38	0.021	0.97
XITN	0.16	4.50	0.73	0.14	0.10	0.29	0.003	0.90

Average values from independent duplicate cultivations. In all cases, the standard error is less than 3%.

^a^Maximum specific growth rate (h^-1^).

^b^Specific volumetric rate of glucose or xylose (g L^-1^ h^-1^).

^c^Biomass, ethanol or glycerol yield on consumed sugars (g g^-1^ sugar).

### NADH oxidase expression decreases xylitol and glycerol accumulation in recombinant XR-XDH strains

**Figure 4:**
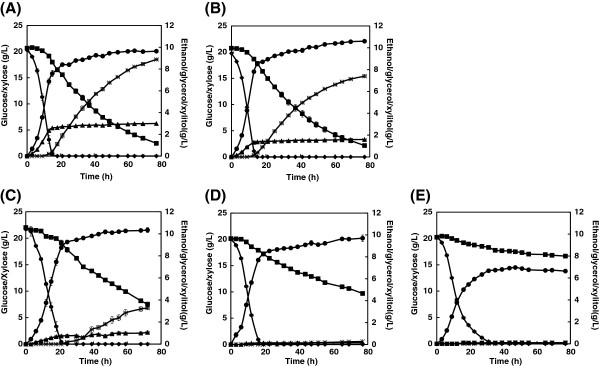
**Aerobic batch cultivations of recombinant XR-XDH strains with a mixture of 20 g/L glucose and 20 g/L xylose.** The experiments were performed in duplicate. **(A)**, XRCO; **(B)**, XRGN; **(C)**, XRTN2; **(D)**, XRHN; and **(E)**, XRTN. Symbols: ◆, glucose; ■, xylose; ●, ethanol; ▲, glycerol; ×, xylitol. Measurements are the average value ± standard error from independent duplicate cultivations.

We also investigated the effect of NADH oxidase expression in recombinant XR-XDH strains (Figure 3 and Table 3). Controlling NADH oxidase expression under the *GPD2* promoter was much less effective than in the recombinant XI strains; the glycerol and xylitol yields were reduced by only 50% and 15%, respectively (Figure 4B and Table 3). This suggested that the NADH oxidation level in XRGN was not sufficiently high to suppress byproduct accumulation. However, increased expression under the *TEF1* promoter led to a large reduction of ethanol yield and inhibited glucose or xylose consumption, indicating that NADH oxidation level in XRTN were prohibitively high (Figure 4E and Table 3). Therefore, we introduced two medium levels of *noxE* expression, under the control of the *HXK2* promoter in the 2 μ plasmid (strain XRHN) and the *TEF1* promoter in the centromeric plasmid (strain XRTN2). The transcription levels of the *noxE* gene and the specific enzyme activities of NADH oxidase were then compared. The relative transcriptions of *noxE* in XITN and XRHN were about 7 fold and 1 fold higher than in XRTN2, respectively, while the relative transcription of *noxE* in XIGN was slightly lower than in XRTN2 (Figure 2A). The specific enzyme activities of NADH oxidase in XRTN, XRHN, XRTN2 and XIGN were 1.92, 0.57, 0.17 and 0.05 U mg^-1^ protein respectively (Table 1). Relative to XRCO, the intracellular NADH/NAD^+^ ratio decreased by 66%, 39%, 33% and 21% in XRTN, XRHN, XRTN2 and XRGN, respectively, when NADH oxidase was expressed (Figure 2B).

**Table 3:** **Physiological parameters of recombinant *S. cerevisiae *** **strains from batch cultivations on glucose and xylose**

**Strain**	**μ**_**max **_**(h**^**-1**^**)**^**a**^	**Consumed xylose in 78 h (g L**^**-1**^**)**	**Sugar consumption rate (g L**^**-1**^ **h**^**-1**^**)**^**b**^		**Yield on sugars (g g**^**-1**^**)**^**c**^			**Xylitol yield (g g**^**-1**^**)**^**d**^	**Carbon balance**
			**Glucose**	**Xylose**	**Biomass**	**Ethanol**	**Glycerol**		
XRCO	0.17	18.53	1.37	0.37	0.085	0.35	0.121	0.53	0.90
XRGN	0.18	18.47	1.39	0.37	0.094	0.37	0.035	0.45	0.86
XRTN2	0.16	13.79	1.20	0.29	0.080	0.36	0.023	0.21	0.82
XRHN	0.17	10.69	1.18	0.24	0.098	0.35	0	0.03	0.91
XRTN	0.15	3.31	0.98	0.08	0.094	0.29	0	0	0.84

Shown are the average values from independent duplicate cultivations. In all cases, the standard error is less than 3%.

^a^ Maximum specific growth rate (h^-1^).

^b^ Specific volumetric rate of glucose or xylose (g L^-1^ h^-1^).

^c^ Biomass, ethanol or glycerol yield from consumed sugars (g g^-1^ sugar).

^d^ Xylitol yield on xylose (g g^-1^ xylose).

As shown in Figure 4, although glycerol and xylitol accumulation was completely prevented in XRHN, the specific xylose consumption rate was 35% lower than in the control strain XRCO (Figure 4D and Table 3). However, in strain XRTN2, the glycerol yield and xylitol accumulation were reduced by 83% and 60% respectively, but the specific xylose consumption rate was 21% lower than in XRCO (Figure 4C and Table 3). Relative to the control strain, byproduct formation was reduced in strain XRTN2 without significantly affecting xylose metabolism. However, none of the recombinant strains improved the ethanol yield relative to XRCO. This indicates that, although NADH oxidase decreased glycerol and xylitol production in recombinant XR-XDH strains, it did not increase the ethanol production in recombinant XR-XDH pathway strains.

## Discussion

In the presence of oxygen, water-forming NADH oxidase can oxidize cytosolic NADH to NAD^+^, with simultaneous reduction of O_2_ to H_2_O. Although NADH oxidase expression is known to reduce xylitol production during xylose metabolism, it requires aerobic cultivation combined with oxygen-limited fermentation; otherwise the enzyme cannot function [20]. However, aerobic cultivation reduces ethanol production. Our respiratory-deficient strain can efficiently generate ethanol from aerobic glucose and xylose cultivations. As such, it provides a solid platform for NADH oxidase expression [12].

During the catabolism of glucose or xylose through the XI pathway, *S. cerevisiae* yields a surplus of cytosolic NADH, mainly through biosynthesis of proteins, nucleic acids and lipids [38,39]. Although cytosolic NADH is required for converting acetaldehyde to ethanol, ethanol production is a redox-neutral process, hence does not contribute to surplus NADH oxidation. The additional NADH generated from catabolism is oxidized via the mitochondrial electron transport chain and glycerol production. In the recombinant XR-XDH strains, the cofactor imbalance between XR and XDH and the increase in cytosolic NADH generated by XDH lead to xylitol accumulation and low ethanol conversion. The difference in cytosolic NADH accumulation between these two situations alters the requirement for NADH oxidase. Indeed, we observed that the recombinant XR-XDH strains required higher NADH oxidase expression than the recombinant XI strain to eliminate glycerol and xylitol.

Glycerol production is consequent to accumulation of excess NADH in the cytosol [40]. The glycerol formation reaction is mediated by glycerol 3-phosphate dehydrogenase (encoded by genes *GPD1* and *GPD2*). Gpd1p is involved in osmosensing and regulation, while Gpd2p is responsible for reoxidation of excess cytosolic NADH [41]. When NADH oxidase is expressed under the control of the *GPD2* promoter, the enzyme competes to oxidize excess NADH that is normally reoxidized by glycerol 3-phosphate dehydrogenase during glycerol production. Therefore, NADH oxidase could be fine-tuned in strain XIGN to eliminate glycerol production without draining the NADH pool. Conversely, when NADH oxidase was expressed under the control of the *GPD2* promoter in the recombinant XR-XDH strains, the excess cytosolic NADH production reduced the effect of the enzyme. Consequently, the expressed NADH oxidase was insufficient to eliminate both xylitol and glycerol, and higher expression was necessary. Although xylitol and glycerol production was completely inhibited in the XRHN (in which NADH oxidase expression is controlled by the *HXK2* promoter on the 2 μ plasmid), xylose consumption by this strain was decreased by 35% relative to the control strain. XRTN2 (in which NADH oxidase expression is controlled by the *TEF1* promoter on the centromeric plasmid) demonstrated lower NADH oxidase activity than XRHN, and yielded low xylitol and glycerol levels without significantly compromising xylose consumption. However, xylose metabolism was decreased by 21% in this strain and some xylitol accumulation was observed. This indicates that NADH oxidase expression in XRTN2 was insufficient to eliminate byproducts, but was sufficiently high to affect substrate metabolism.

Comparing the optimal modified strains in both pathways, the recombinant XI strain XIGN accumulated little byproduct, and demonstrated a higher xylose consumption rate and ethanol yield than the recombinant XR-XDH strain XRTN2. Therefore, the performance of the recombinant XI strain surpassed that of the recombinant XR-XDH strain. Additionally, our recombinant rumen XI strains XIGN performed similarly to an *S. cerevisiae* strain expressing *Piromyces* sp. *xylA*[8,11,13] in terms of ethanol production, but accumulated considerably less glycerol. Consequently, the recombinant XI strain emerges as a preferable choice for lignocellulosic feedstock utilization.

## Conclusion

In this study, we investigated the impact of NADH oxidase on two xylose metabolism pathways: the cofactor independent XI pathway and the cofactor dependent XR and XDH pathway. According to our results, NADH oxidase expression decreases glycerol and xylitol accumulation in respiration-deficient xylose-metabolizing *S. cerevisiae*. By controlling the promoter strength of *noxE* gene and the plasmid copy number, we obtained an efficient xylose-metabolizing strain that accumulated little byproduct. Such fine-tuned cofactor engineering is an attractive strategy for bioprocessing, and is applicable to production of other redox dependent metabolites.

## Methods

### Plasmids and strains construction

The primers used in this study are listed in Table 4. The *noxE* gene (GenBank Accession no. 4796799) was amplified from the genomic DNA of *Lactococcus lactis subsp.* MG1363. A 2 μ plasmid pYX242-WS (constructed from pYX242 by inserting the *TEF1* promoter in front of the *polyA* terminator) and a centromeric plasmid pRS315 were used for *noxE* gene expression. Ligation was performed using T4 DNA ligase or a one-step enzymatic DNA assembly protocol [42]. The *noxE* gene was constructed under the control of *TEF1*, *GPD2* or *HXK2* promoters and *polyA* terminator in pYX242-WS [42], or the *TEF1* promoter and *polyA* terminator in pRS315 (Table 5). The genetic reference strain was BSPX042 (*ura3-52*, *XKS1::loxP-TEF1p*, gre3(-241,+338)::*TPI1p-RKI1-RKI1t-PGK1p-TAL1-TAL1t-FBA1p-TKL1-TKL1t-ADH1p-RPE1-RPE1t-loxP*, *cox4::loxP*, adaptive evolution) as described in our previous work [28] (Table 5). The recombinant yeast strains and plasmids used in this study are listed in Table 5. The leucine auxotrophic strain, obtained by deleting the *LEU2* gene in BSPX042, was named BSLS000. The recombinant XR-XDH and XI strains were constructed by transforming the pJX1 (*XYL1* and *XYL2* genes from S. stipitis) and pJX7 (*xylA* gene from bovine rumen, GenBank Accession no. JF496707) plasmids, respectively, into BSLS000. The empty plasmid pYX242-WS or plasmids containing the *noxE* gene was then transformed into the recombinant XR-XDH or XI strains, to construct XICO (XI, pYX242-WS), XITN (XI, pYX242-TEF1nox), XIGN (XI, pYX242-GPD2nox), XRCO (XR-XDH, pYX242-WS), XRTN (XR-XDH, pYX242- TEF1nox), XRGN (XR-XDH, pYX242-GPD2nox), XRHN (XR-XDH, pYX242-HXK2nox) and XRTN2 (XR-XDH, pRS315-TEF1nox) (Table 5).

**Table 4:** Primers used in this study

**Primer name**	**Sequence (5′ → 3′)**	**Template**	**Purpose**
Leu2 up	ATGTCTGCCCCTAAGAAGATCGTCGTTTTGCCAGGTGACAGCTGAAGCTTCGTACGCTG	pUG6	*LEU2* gene deletion
Leu2 down	CACCAGTTCTGATACCTGCATCCAAAACCTTTTTAACTGATAGGCCACTAGTGGATCTG	pUG6	*LEU2* gene deletion
TEF1W up	CCCAAGCTTCACAATGCATACTTTGTACGTT^a^	BSPX042 chromosome	pYX242-WS
TEF1W down	GCGCGTCGACTTGTAATTAAAACTTAGATTAG^b^	BSPX042 chromosome	pYX242-WS
noxE up	ACGCGTCGACATGAAAATCGTA^c^	*L. lactis* chromosome	pYX242- TEF1nox
noxE down	GTCCGAGCTCTTATTTGGCATTC^d^	*L. lactis* chromosome	pYX242- TEF1nox
GPD2p up	TTTAATAACTCGAAAATTCTGCGTTCGTTAAAGCTTCGACATATCTATTATAGTGGGGAGA	BSPX042 chromosome	pYX242- GPD2nox
GPD2p down	GCCTGCGTGGTTTGTACCGATAACTACGATTTTCATGTCGACACGACTAGTGACAGCAAGCATT	BSPX042 chromosome	pYX242- GPD2nox
HXK2p up	TAAGTTTAATAACTCGAAAATTCTGCGTTCGTTAAAGCTTTTGAAAAAAAGTGCGGGGC	BSPX042 chromosome	pYX242-HXK2nox
HXK2p down	CTGCGTGGTTTGTACCGATAACTACGATTTTCATGTCGACTTTATTTAATTAGCGTACT	BSPX042 chromosome	pYX242-HXK2nox
TEF1p up	TGGAGCTCCACCGCGGTGGCGGCCGCTCTAGAACTAGTGGATCCCACAATGCATACTTT	BSPX042 chromosome	pRS315-TEF1nox
TEF1p down	GGGTACCGGGCCCCCCCTCGAGGTCGACGGTATCGATAAGCTTAGCCGGCGAACGTGGC	BSPX042 chromosome	pRS315-TEF1nox
ACT1qPCR up	CAAACCGCTGCTCAATCTTC	cDNA	qPCR
ACT1qPCR down	AGTTTGGTCAATACCGGCAG	cDNA	qPCR
NOXqPCR up	TACTGCCAACAGTGCCTTGG	cDNA	qPCR
NOXqPCR down	TTCCTGACCGAACAGCGTTT	cDNA	qPCR

^a^underline: *Hind* III restriction site used for ligation.

^b^underline: *Sal*I restriction site used for ligation.

^c^underline: *Sal*I restriction site used for ligation.

^d^underline: *Sac*I restriction site used for ligation.

**Table 5:** Plasmids and strains used in this study

**Strain/plasmid**	**Genotype/properties**	**Source of reference**
*S. cerevisiae*		
CEN.PK113-5D	*MATa SUC2 MAL8C ura3-52*	Peter Kötter
BSPX042	CEN.PK113-5D, XKS1::loxP-TEF1p, gre3(-241, +338) ::*TPI1p-RKI1-RKI1t-PGK1p-TAL1-TAL1t-FBA1p-TKL1-TKL1t-ADH1p-RPE1-RPE1t-loxP*, *cox4::loxP*, adaptive evolution, *ura3-52*	[28]
BSLS000	BSPX042, *leu2::loxp-KanMX-loxp*	This study
XICO	BSLS000, pJX7&pYX242-WS	This study
XITN	BSLS000, pJX7&pYX242-TEF1nox	This study
XIGN	BSLS000, pJX7&pYX242-GPD2nox	This study
XRCO	BSLS000, pJX1&pYX242-WS	This study
XRTN	BSLS000, pJX1&pYX242-TEF1nox	This study
XRGN	BSLS000, pJX1&pYX242-GPD2nox	This study
XRHN	BSLS000, pJX1&pYX242-HXK2nox	This study
XRTN2	BSLS000, pJX1&pRS315-TEF1nox	This study
Plasmids		
pYX242-WS	2 μ plasmid with *TEF1* promoter and Poly A terminator, *LEU2* marker	This study
pRS315	Centromeric plasmid with *LEU2* marker	ATCC77144
pYX242-TEF1nox	pYX242-WS, *noxE* gene with *TEF1* promoter, *LEU2*	This study
pYX242-GPD2nox	pYX242-WS, *noxE* gene with *GPD2* promoter, *LEU2*	This study
pYX242-HXK2nox	pYX242-WS, *noxE* gene with *HXK2* promoter, *LEU2*	This study
pRS315-TEF1nox	pRS315, *noxE* gene with *TEF1* promoter, *LEU2*	This study
pJX1	YCplac33, *XYL1* with *TEF1* promoter, *XYL2* with *TDH3* promoter	[12]
pJX7	YEplac195, 2 μ, *Ru-xylA* with *TEF1* promoter, *URA3*	[28]

### Growth conditions

*E. coli* recombinant cells were grown in Luria-Bertani medium (5 g/L yeast extract, 10 g/L tryptone, 10 g/L NaCl, pH 7.0) in the presence of ampicillin (100 mg/L) at 37°C. Recombinant strains of *S. cerevisiae* were cultured in SD medium (1.7 g/L yeast nitrogen base, 5 g/L (NH_4_)_2_SO_4_) with amino acid lacking uracil and/or leucine and 20 g/L glucose at 30°C, with rotation at 200 rpm.

### Batch cultivations

Preculture was prepared by inoculating the strains in 400 mL SD medium containing 20 g/L glucose in 1 L shake flasks. The batch cultivations were carried out in 1.4 L fermenters (Infors AG, Switzerland) with a working volume of 1 L and controlled at 30°C, 600 rpm and 0.1 vvm. The pH was maintained at 5.0 by automatic addition of 1 M H_3_PO_4_ or 1 M NaOH. The quantity of CO_2_ and O_2_ in the exhaust gases was measured with a gas analyzer. All cultivations were carried out in defined medium containing 5 g/L (NH_4_)_2_SO_4_, 1.7 g/L yeast nitrogen base, 20 g/L glucose and 20 g/L xylose. The initial OD_600_ was adjusted to 1. All cultivations were performed in duplicate.

### Extracellular metabolite analysis

Samples from the fermenters were centrifuged and filtered through a 0.45 μm pore size syringe filter and frozen at -20°C for subsequent analysis. Concentrations of glucose, xylose, xylitol, glycerol, ethanol and acetic acid were analyzed by HPLC. The separation column was an Aminex HPX-87H ion exchange column (Bio-Rad, Hercules, USA) operating at 45°C with mobile phase 5 mM H_2_SO_4_ at a flow rate of 0.6 mL/min. The peaks were detected by RI and UV detectors.

### Cell mass determination

The optical density of the culture at 600 nm was measured in a spectrophotometer (Eppendorf AG, 22331 Hamburg, Germany). The dry cell weight (DCW) was measured by filtering a known volume of the culture through a pre-dried and pre-weighed nitrocellulose filter (pore size 0*.*45 μm). The filters were dried at 105°C until their weight had stabilized, and re-weighed. The dry cell weight was the weight difference between the filter membrane with dried cells and the membrane without cells. The dry cell weight was estimated from the measured OD_600_-dry weight correlation, where 1 g/L biomass equals 0.246× (OD_600_) - 0.0012.

### NADH oxidase activity measurement

Samples were harvested during the mid-exponential phase of the cultivation and centrifuged immediately (4,000 *g* at 1°C for 5 min). Cell-free extracts were prepared using a Fast Prep cell homogenizer. NADH oxidase activity was assayed spectrophotometrically in 50 mM potassium phosphate buffer (pH 7.0), 0.3 mM β-NADH and 0.3 mM EDTA at 340 nm, as previously described [43]. Protein concentrations were measured following the Bradford method. One unit (U) of enzyme activity was defined as the oxidation of 1.0 μmol NADH per min.

### NAD^+^ and NADH quantification

Samples were taken and quenched in 30 mL pure methanol in pre-weighed tubes maintained at -40°C. The cells were then collected by centrifugation at -20°C at 12000 *g* for 5 minutes. Next, 1 ml of 17% (v/v) alcoholic 1 M KOH (for NADH extraction) or 1 ml 35% (v/v) HClO_4_ (for NAD^+^ extraction) was added to the cell pellet. The extracts were frozen by liquid nitrogen, thawed and then neutralized by adding 2 M HCl (for NADH extraction) or 2 M KOH (for NAD^+^ extraction). The cellular debris was removed by centrifuging at 12,000 *g* for 5 min. Supernatants were transferred to new tubes and stored at -80°C. Intracellular NAD(H) concentrations were determined by HPLC as previously described [44].

### Real-time quantitative PCR (qPCR)

Total RNA was isolated using UNIQ-10 spin column RNA extraction kits (Sangon Biological Engineering, China). The first cDNA strand was synthesized using the PrimeScript™ RT Reagent Kit (TaKaRa, Japan) and was used for qPCR amplification in the Light Cycle PCR System (SYBR Green Real-time PCR Master Mix, Japan). The reference gene was *ACT1*. The gene-specific primers are listed in Table 4.

## Abbreviations

XI: Xylose isomerase; XR: Xylose reductase; XDH: Xylitol dehydrogenase; NADH: Reduced form of nicotinamide adenine dinucleotide; DHAP: Dihydroxyacetone phosphate; GPD: Glycerol-3-phosphate dehydrogenase; GPP: Glycerol-3-phosphate phosphatase; EDTA: Ethylenediaminetetraacetic acid.

## Competing interests

The authors declare that they have no competing interests.

## Authors’ contributions

JH designed the study, performed the data analysis and wrote the manuscript. SF performed the experiments and edited the manuscript. WC and LX performed some experiments. YS participated in design and drafted the manuscript. BX participated in the design and coordination and drafted the manuscript. All authors read and approved the final manuscript.
